# Glueless and Sutureless Multi-Layer Amniotic Membrane Transplantation in a Patient With Pending Corneal Perforation

**DOI:** 10.7759/cureus.16678

**Published:** 2021-07-27

**Authors:** Anastasios Lavaris, Mohamed F M Elanwar, Motasim Al-Zyiadi, Paraskevi T Xanthopoulou, Nick Kopsachilis

**Affiliations:** 1 Ophthalmology, East Kent Hospitals University NHS Foundation Trust, Canterbury, GBR

**Keywords:** amniotic membrane, ocular graft-versus-host-disease, corneal thinning, ocular surface disease, corneal perforation

## Abstract

Ocular graft-versus-host disease (GVHD) is a severe complication of allogenic hematopoietic stem cell transplantation (HSCT). It is a term used to describe a spectrum of signs and symptoms including ocular surface inflammation, dry eye syndrome, lacrimal and meibomian gland dysfunction. We present a case of a 73-year-old man with chronic myeloblastic leukaemia and chronic GVHD. On examination, severe corneal thinning was detected in his left eye. We performed multi-layer amniotic membrane patching of the affected area, in an ambulatory setting, without using sutures or glue, but only a bandage contact lens to keep amniotic membranes attached. Three months post-amniotic-membrane-patching symptoms improved, corneal integrity was maintained, and corneal thickness increased significantly. Multi-layer amniotic membrane patching without glue and sutures may be sufficient enough to prevent further deterioration of corneal thinning and can be safely performed as an outpatient procedure, reducing the need for tectonic corneal transplantation.

## Introduction

Haematopoietic stem cell transplantation (HSCT), previously referred to as bone marrow transplantation, can be used in the treatment of immune haematologic disorders and haematologic malignancies. The source of donor cells can be the patient (autologous) or another individual (allogeneic) [1]. Despite the benefits of allogeneic HSCT in treating haematological malignancies, graft-versus-host disease (GVHD) is a serious post-transplantation complication. In GVHD, donor cells mount an immunologic attack against the host, mainly targeting the skin, gastrointestinal system, mouth, liver, lungs and eyes [2]. Ocular GVHD may affect up to 60% of patients receiving allogeneic HSCT and is mainly characterised by ocular surface and lacrimal glands inflammation and scarring, dry eye disease and meibomian gland dysfunction [3]. Dry eye syndrome (DES) is the most common ocular complication in patients undergoing HSCT [4]. Artificial tears, punctal plugs, topical corticosteroids and immunosuppressants are the main therapeutic options in treatment of ocular GVHD and associated DES [5]. The efficacy of amniotic membrane (AM) in treating severe immune or non-immune related DES, chemical and thermal injuries, Stevens-Johnson syndrome, immune-related peripheral ulcerative keratitis, necrotizing scleritis and limbal stem cell deficiency has been demonstrated. AM has anti-inflammatory, anti-microbial, anti-scarring, and anti-angiogenic effects when applied on the ocular surface. While sutured or glued AM patching has been used in treating corneal thinning and severe DES in cases of ocular GVHD, there are no reported cases in the literature of using a sutureless and glueless technique of multi-layer AM patching [5-8]; therefore we are presenting a case where this technique had a satisfying outcome.

## Case presentation

A 73-year-old man, with a known history of ocular graft-versus-host disease and a background of chronic myeloblastic leukaemia on oral ponatinib, who had undergone two bone marrow transplantations in the past (the latter being two years ago), was referred to our department by his optician. He was complaining of ''exacerbation of his dry eye disease'', with significant pain and photophobia in his left eye. His topical medication for dry eyes included preservative-free artificial tears used four times per day in both eyes and topical ciclosporin 1mg/ml used twice per day in both eyes. His medication also included amlodipine and perindopril for arterial hypertension as well as oral zopiclone.

**Figure 1:**
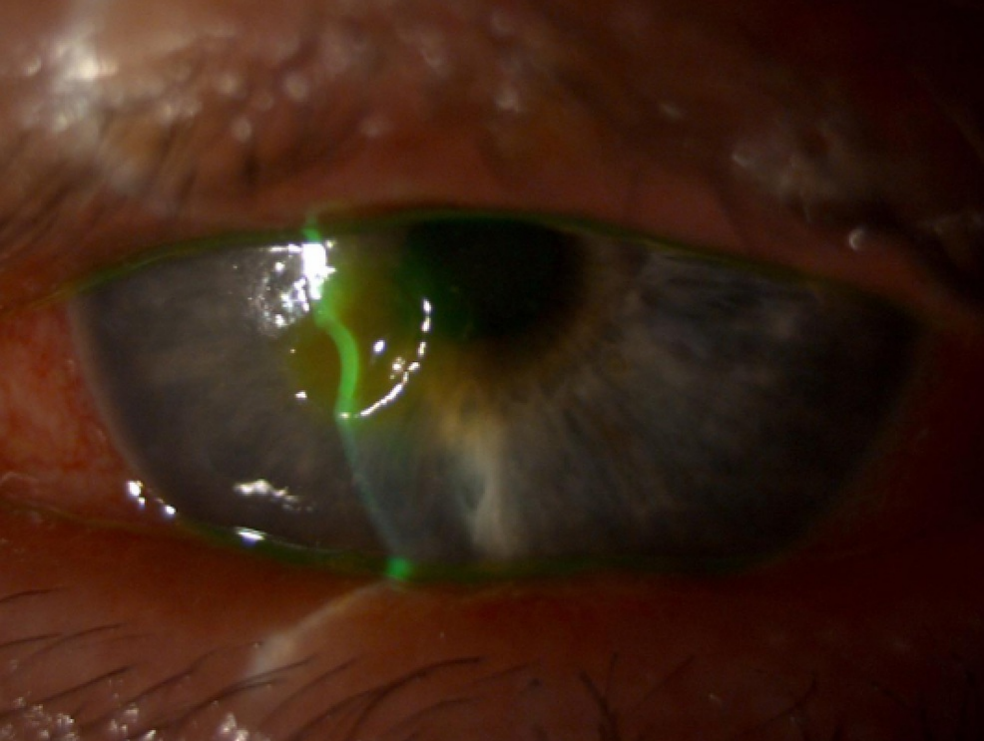
Slit lamp image of the left eye, prior to amniotic membrane transplantation, demonstrating marked paracentral stromal thinning

On examination, marked paracentral corneal stromal thinning was detected in his left eye (Figure 1). Neither infiltration nor anterior chamber reaction was present in either eye. Fundus examination was unremarkable in both eyes.

**Figure 2:**
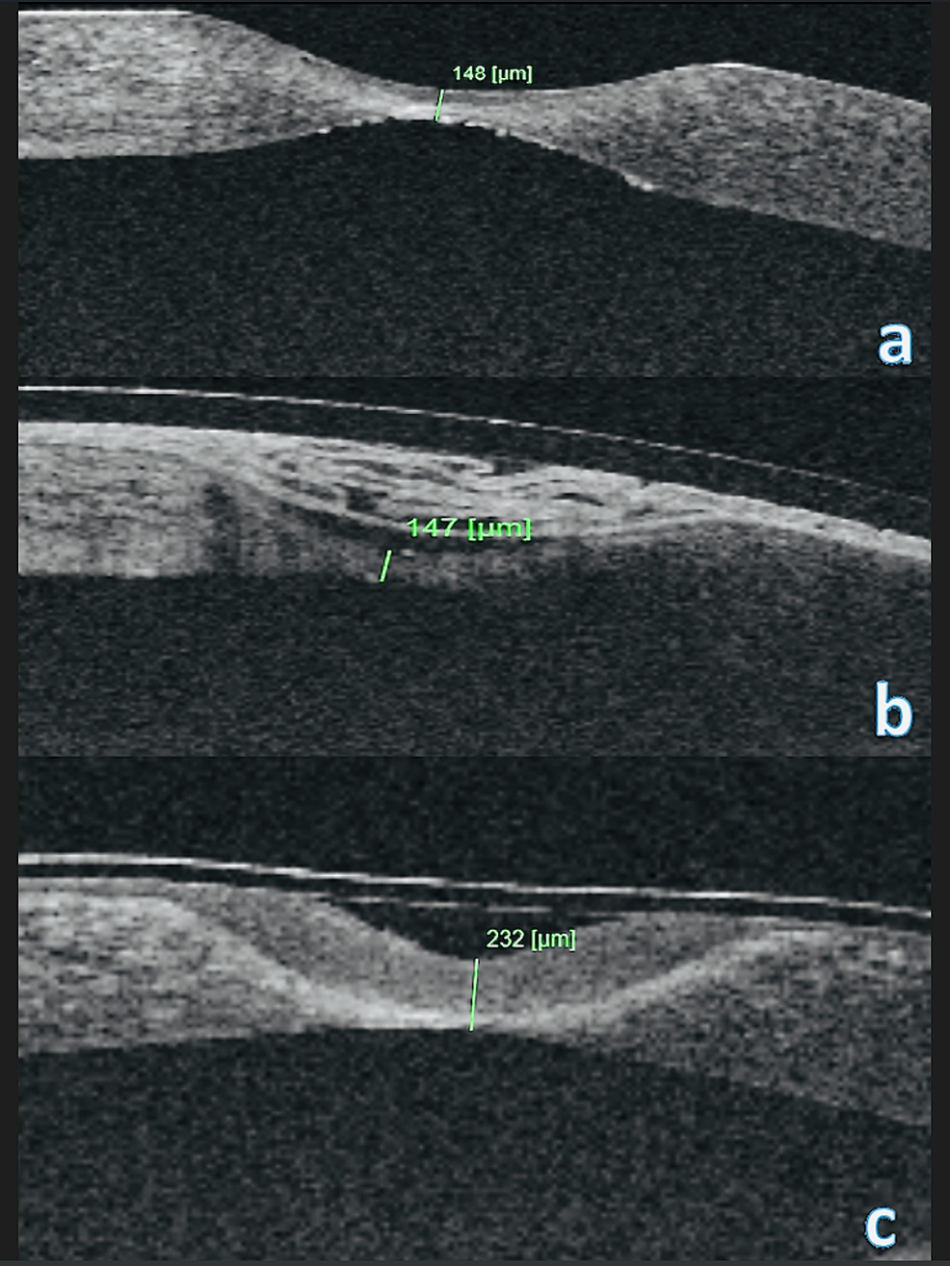
Anterior segment optical coherence tomography (OCT) and thickness measurement pre-operatively (a), at 1 month (b) and 2 months (c) following the procedure

A sutureless and glueless technique of multi-layer amniotic membrane patching of the affected area was performed in his left eye, using a novel, low-temperature, vacuum-dried amniotic membrane (Omnigen®; NuVision Biotherapies Ltd., Nottingham, UK). One amniotic membrane graft was folded and facilitated as material to fill the stromal thinning area and a second graft was used, stromal side down, to cover the first graft and the cornea. A large (20mm diameter bandage contact lens (Omnilenz®; NuVision Biotherapies Ltd.) was fitted to the left eye. Following this procedure, a regimen of topical ciclosporin 1mg/ml, preservative-free dexamethasone 0.1%, topical preservative-free chloramphenicol 0.5% and preservative-free cyclopentolate 1% was prescribed and follow up was arranged one week, one month, two months and three months following this procedure. Figures 2a, 2b, 2c demonstrate anterior segment optical coherence tomography (OCT) image and corneal thickness measurement pre-operatively, at one month and two months following the procedure, respectively. One can see the increased thickness of the epithelial layer and the overall improved structural integrity of the cornea.

## Discussion

The armamentarium of available treatment options of DES and corneal thinning in ocular GVHD remains limited. Conventional treatment with tear supplements and bandage contact lenses is inadequate to reverse the immunologic process of GVHD and to prevent a corneal perforation.

Chronic use of topical corticosteroids has the known risks of corneal infection, cataractogenesis, ocular hypertension and glaucoma. Topical application of autologous serum drops may restore the ocular surface, but numerous disadvantages including reduced stability, risk of contamination and infections have been reported [9]. The application of sutured, cryopreserved AM in the management of ocular GVHD has been described [6,10], but it has to be performed in an operating theatre setting.

## Conclusions

In this regard, we underline the efficacy of sutureless and glueless vacuum-dried amniotic membrane application, as it can be easily performed in the clinic. The AM can integrate into the host cornea by formation of hemidesmosomes that provide anchoring for the regenerating epithelium.

This outpatient clinic-based procedure, which eliminates the risk of surgical complications and suture-induced inflammation, appears to be capable of preventing irreversible damage, including corneal perforation and the need for further surgical treatment.
